# Genomic Features of the Bundle-Forming Heliobacterium *Heliophilum fasciatum*

**DOI:** 10.3390/microorganisms10050869

**Published:** 2022-04-21

**Authors:** Kelly S. Bender, Michael T. Madigan, Kyleigh L. Williamson, Marisa H. Mayer, Mary N. Parenteau, Linda L. Jahnke, Paula V. Welander, Sophia A. Sanguedolce, Abigail C. Brown, W. Matthew Sattley

**Affiliations:** 1 Microbiology Program, School of Biological Sciences, Southern Illinois University, Carbondale, IL 62901, USA; benderk@siu.edu (K.S.B.); madigan@siu.edu (M.T.M.); kyleigh.williamson@siu.edu (K.L.W.); 2 Exobiology Branch, NASA Ames Research Center, Moffett Field, CA 94035, USA; marisa.h.mayer@nasa.gov (M.H.M.); mary.n.parenteau@nasa.gov (M.N.P.); linda.l.jahnke@nasa.gov (L.L.J.); 3 Department of Earth System Science, Stanford University, Stanford, CA 94305, USA; welander@stanford.edu; 4 Division of Natural Sciences, Indiana Wesleyan University, Marion, IN 46953, USA; sophia.sanguedolce@myemail.indwes.edu (S.A.S.); abbey.brown2@myemail.indwes.edu (A.C.B.)

**Keywords:** anoxygenic phototrophs, heliobacteria, bacteriochlorophyll *g*, *Heliophilum fasciatum*, genome sequence

## Abstract

Eight species of heliobacteria have had their genomes sequenced. However, only two of these genomes have been analyzed in detail, those from the thermophilic *Heliomicrobium (Hmi.) modesticaldum* and the alkaliphilic *Heliorestis (Hrs.) convoluta*. Here we present analyses of the draft genome sequence of a species of heliobacterium that grows optimally at a moderate temperature and neutral pH. The organism, *Heliophilum (Hph.) fasciatum*, is phylogenetically unique among cultured heliobacteria and was isolated from rice soil, a common habitat for heliobacteria. The *Hph. fasciatum* genome contains 3.14 Mbp—similar to that of other reported heliobacteria—but has a G+C base ratio that lies between that of *Hmi. modesticaldum* and *Hrs. convoluta*. Many of the genomic features of *Hmi. modesticaldum* and *Hrs. convoluta*, such as the absence of genes encoding autotrophic pathways, the presence of a superoperonal cluster of photosynthesis-related genes, and genes encoding endospore-specific proteins, are also characteristic of the *Hph. fasciatum* genome. However, despite the fact that *Hph. fasciatum* is diazotrophic, classical *nif* genes encoding the alpha and beta subunits of dinitrogenase (*nifDK*) present in other heliobacteria could not be identified. Instead, genes encoding several highly divergent NifDK homologs were present, at least one of which likely encodes a functional dinitrogenase and another a methylthio-alkane reductase (MarDK) for sulfur assimilation. A classical NifH (dinitrogenase reductase) homolog was also absent in *Hph. fasciatum*, but a related protein was identified that likely carries out this function as well as electron delivery to MarDK. The N_2_-fixing system of *Hph. fasciatum* is therefore distinct from that of other heliobacteria and may have unusual properties.

## 1. Introduction

*Heliobacteriaceae* comprise a family of anoxygenic phototrophic bacteria phylogenetically and physiologically distinct from purple and green bacteria [1,2]. Unlike the latter two groups, heliobacteria do not contain bacteriochlorophyll (Bchl) *a*, *b*, *c*, *d*, or *e*, but instead, produce Bchl *g* as their main pigment, a bacteriochlorophyll structurally most closely related to green plant chlorophyll *a* [3]. In addition, heliobacteria also differ from purple and green bacteria in that heliobacteria are of gram-positive lineage, are obligate anaerobes, lack all known biochemical pathways for autotrophic growth, and produce heat-resistant endospores [4,5,6].

**Figure 1:**
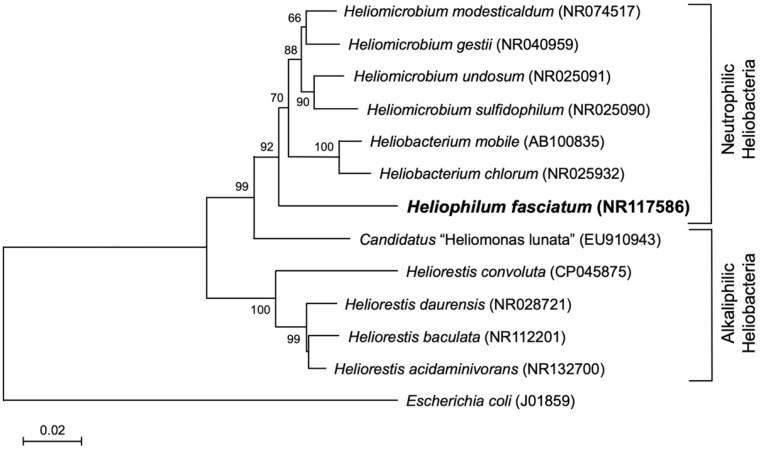
Phylogeny of *Heliophilum fasciatum* based on 16S ribosomal RNA gene sequence analysis. The multiple alignments included 16S rRNA gene sequences from all cultured type strains of the *Heliobacteriaceae*, as well as *Candidatus* “Heliomonas lunata” and the outgroup species *Escherichia coli*. The neighbor-joining tree was constructed from 1344 nucleotide positions. The scale bar indicates the number of base substitutions per site. Bootstrap values > 50 (500 replicates) are indicated at each node, and GenBank accession numbers are shown in parentheses.

Several species of heliobacteria have been isolated since the discovery of *Heliobacterium (Hbt.) chlorum* by Gest and Favinger in the early 1980s [1,7] reviewed in [2,4,8], and described species form two broad groups on the basis of their pH optima for growth: neutrophilic species, with pH optima near 7, and alkaliphilic species, with pH optima near 9. This physiological dichotomy of heliobacteria is mirrored in their phylogeny (Figure 1). Although several species of heliobacteria have had their genomes sequenced, thus far only two species—both extremophiles—have had their genomes analyzed in detail. These include the thermophilic (and neutrophilic) species *Heliomicrobium* (previously *Heliobacterium*) [8] *modesticaldum* [9,10,11] and the alkaliphilic species *Heliorestis convoluta* [12,13]. These studies have provided the genomic support for the aforementioned unusual physiological and biochemical features of the heliobacteria and confirmed that all characterized species (with the exception of *Candidatus* “Heliomonas lunata” [14]) are robust diazotrophs [15,16].

**Figure 2:**
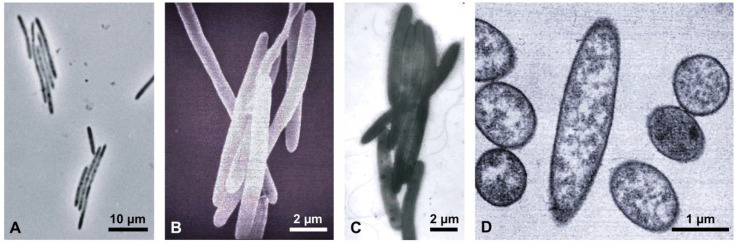
*Heliophilum fasciatum*. (**A**) Phase-contrast micrograph of cells formed into motile bundles. (**B**) Scanning electron micrograph of a cell bundle. (**C**) Negatively stained transmission electron micrograph with visible flagella. (**D**) Thin sectioned transmission electron micrograph showing absence of internal photosynthetic membranes, typical of the heliobacteria.

In 1996, Ormerod et al. [16] described a new genus of bundle-forming heliobacteria in which a raft of apparently loosely attached rod-shaped cells displayed swimming motility as a unit (Figure 2). Besides exhibiting the usual assortment of heliobacterial physiological properties [5], this organism, named *Heliophilum fasciatum* (“sun-loving bundle-forming heliobacterium”), differed in several respects from *Hmi. modesticaldum* and *Hrs. convoluta*, most notably in its habitat and phylogenetic status (Table 1). By 16S rRNA gene sequencing criteria, *Hph. fasciatum* lies basal to all known neutrophilic heliobacteria and can be considered a “bridge” species between neutrophilic and alkaliphilic clades (Figure 1). All three of these heliobacteria assimilate acetate and pyruvate during photoheterotrophic growth and only *Hrs. convoluta* is unable to grow fermentatively in darkness (Table 1). In addition, each of these three species is morphologically distinct, shows distinct pH and temperature optima for growth, and was isolated from geographically well separated and geochemically distinct habitats (Table 1 and Table 2).

Despite the fact that *Hph. fasciatum* is phylogenetically and phenotypically distinct from *Hmi. modesticaldum* and *Hrs. convoluta* (Figure 1 and Table 1), this species has remained in the background of heliobacterial research until its genome was recently sequenced as part of a systematic study of the heliobacteria to support taxonomic revisions in the family [8]. Genomic studies of heliobacteria have lagged behind those of purple and green bacteria, and to date, a total of only eight species of heliobacteria have had their genomes sequenced, six of which only to the draft stage. Sequence data have shown the size of heliobacterial genomes to be quite variable, as the largest heliobacterial genome encodes more than a thousand additional proteins than does the smallest (Table 2). In a comparative study of the genomes of the eight species of heliobacteria listed in Table 2 [8], the authors showed that a phylogenetic tree constructed from the genomes of these species closely mirrored the tree constructed from 16S rRNA gene sequences (Figure 1). In addition, the authors used genomic data along with the comparative sequences of a few key genes and operons to propose that three of the species in the genus *Heliobacterium* should be grouped into their own genus, *Heliomicrobium* [8]; these include *Hmi. modesticaldum, Hmi. gestii*, and *Hmi. undosum* (Table 2). Analyses of genes encoding particular photocomplex and endospore proteins supported the taxonomic proposals, as did genes encoding carbon monoxide dehydrogenase, an enzyme previously unsuspected in the heliobacteria [8].

Thus far, detailed analyses of the genomes of only *Hmi. modesticaldum* and *Hrs. convoluta* have been published [10,11,13]. Here we augment these studies with an analysis of the major gene sets of *Hph. fasciatum* using data generated from our own sequencing project. Our results focus on genes encoding proteins of major pathways of carbon, nitrogen, and sulfur metabolism, and genes encoding the formation of endospores and flagellar components, comparing them where appropriate with those from other heliobacteria, in particular, the two species with complete genome sequences (Table 1 and Table 2). Our analyses have uncovered several similarities and differences in the genetic capabilities of *Hph. fasciatum* compared with those of other heliobacteria, including in particular the fact that despite its diazotrophic phenotype, *Hph. fasciatum* lacks genes encoding the canonical nitrogenase universally present in all other cultured heliobacteria and in most other anoxygenic phototrophic bacteria.

## 2. Materials and Methods

The *Heliophilum fasciatum* strain Tanzania^T^ was obtained from the Leibniz-Institut DSMZ, Braunschweig, Germany, as DSM 11170^T^. Cells of *Hph. fasciatum* were grown phototrophically in medium PYE [15], and genomic DNA was isolated using the JetFlex™ Genomic DNA Purification Kit (ThermoFisher Cat No. A30700). The genome was sequenced through the U.S. Department of Energy Joint Genome Institute (JGI) Community Science Program. Whole-genome shotgun sequencing was done with Illumina NovaSeq sequencing (library 300 bp), and the resulting fragments were assembled and annotated by the JGI Integrated Microbial Genomes (IMG) annotation pipeline (IMGAP v.5.0.23 with gene calling program CRT 1.8.2). The genome sequence of *Hph. fasciatum* Tanzania^T^ is publically available (listed as *Heliophilum fasciatum* MTM in the JGI/IMG database) under the genome ID 2929297113. The genome of the exact same strain of *Hph. fasciatum* was independently sequenced at JGI by another group [8] and is publicly available as genome ID 2795386140; the latter sequence was also accessioned into Genbank as reference sequence NZ_SLXT00000000.1.

Phylogenetic analyses of 16S ribosomal RNA genes and protein sequences were performed using MEGA version X [18,19]. The *Hph. fasciatum* strain Tanzania^T^ 16S rRNA gene sequence was aligned with corresponding sequences from other heliobacterial type species and *Escherichia coli* (J01859), used as the outgroup to root the phylogenetic tree. The tree was drawn using MEGA X according to the parameters described in the legend in Figure 1. For trees of *nif*-related proteins, *Hph. fasciatum* NflHDK amino acid sequences were used as queries in blastp [20] searches to identify other heliobacteria homologs. The genetic organization of the *Hmi. gestii* strain DSM 11169 *nif* and *anf* loci were obtained from Genbank genome accession PRJNA599378. Protein phylogenetic analyses were performed on a subset of NifHDK homologs previously designated as belonging to nitrogenase-like protein groups I–IV [21,22]. Following ClustalW [23] analysis, the resulting alignments were assembled into maximum-likelihood trees using the LG + G substitution model [24] with 100 replicates. Note that the files for NifD and NifK homologs were concatenated prior to tree assembly.

## 3. Results and Discussion

### 3.1. General Genomic Properties

Table 3 compares genomic statistics of *Hph. fasciatum* strain Tanzania^T^ with those of *Hmi. modesticaldum* and *Hrs. convoluta*. The genome of *Hph. fasciatum* consists of a single chromosome and was sequenced to 477X coverage and assembled to yield a total of 75 contigs. The estimated size of the *Hph. fasciatum* genome is 3,141,306 base pairs, and its G+C base ratio is approximately equidistant from that of *Hrs. convoluta*, whose genome is near the lowest of genomic G+C ratios in heliobacteria, and *Hmi. modesticaldum*, whose genome is near the highest (Table 3). The average nucleotide identity between the three species’ genomes clearly reflected their phylogenetic positions (Figure 1), and the *Hph. fasciatum* genome is clearly the most gene-dense of the three species. Moreover, the percentage of hypothetical proteins encoded by the *Hph. fasciatum* genome is similar to that of *Hmi. modesticaldum*, and both of these were significantly lower than that of *Hrs. convoluta* (Table 3).

### 3.2. Phototrophy

Genes encoding phototrophy in heliobacteria are organized into an assemblage called the photosynthesis gene cluster (PGC) [10,11]. The arrangement of photosynthesis genes in the PGC of all heliobacteria sequenced thus far is nearly identical [8], and this trend extends to *Hph. fasciatum*. As in other heliobacteria, the *Hph. fasciatum bch* genes encoding biosynthetic proteins of the major heliobacterial pigment Bchl *g* were split into two sub-clusters consisting of *bchJGMELNBIDH* and *bchXZY*, with the gene encoding the homodimeric type I (Fe–S type) reaction center protein PshA positioned between *bchG* and *bchM*. Other genes, including those encoding proteins for carotenoid and cofactor biosynthesis and major aspects of cell division and electron transport, were arranged identically to their complements in other sequenced heliobacteria, including *Hmi. modesticaldum* and *Hrs. convoluta* [10,13].

In heliobacteria, the *bchJGMELNBIDH* and *bchXZY* gene clusters are separated by about 20 genes encoding a variety of proteins, most prominently those necessary for cofactor biosynthesis. However, an unusual feature of the PGC of *Hph. fasciatum* was the insertion between the two *bch* gene clusters of 11 additional genes encoding proteins unrelated to photosynthesis. This assortment of genes encodes a variety of proteins including the spore-coat protein CotF (discussed later), carbon monoxide dehydrogenase, a nucleotide-binding protein for DNA uptake, a transcription regulator, and a transposase. Based on the heliobacterial genomes currently available and the phylogenetic divergence of *Hph. fasciatum* from other heliobacteria (Figure 1), this multiple-gene insertion may be unique to the PGC of this particular species.

### 3.3. Central Carbon Metabolism

Although all heliobacteria presumably exhibit a common mechanism for light-harvesting photochemistry (for a recent summary, see Orf and Redding [5]), carbon source utilization varies somewhat among species of *Heliobacteriaceae*. Although a few heliobacteria (e.g., *Hmi. gestii* and *Hmi. modesticaldum*) are able to use certain carbohydrates as a carbon source [16,17], all heliobacteria catabolize pyruvate as a preferred carbon source, and most others can use yeast extract, lactate, or certain fatty acids for photoheterotrophic growth [4]. In addition to the best photoheterotrophic growth on pyruvate or lactate, good growth of *Hph. fasciatum* was demonstrated on acetate or butyrate plus CO_2_, and weaker but measurable growth on ethanol plus CO_2_ [16]. As is true for all other heliobacteria thus far studied, photoautotrophic growth (light/H_2_ + CO_2_) of *Hph. fasciatum* has not been demonstrated, and therefore, as a group, heliobacteria are phototrophic but not photosynthetic in the usual sense of this word since they lack a complete autotrophic pathway. Besides H_2_, photoautotrophic growth of *Hph. fasciatum* using sulfide as an electron donor also was not observed [16]. For *Hph. fasciatum*, sulfide levels above 0.1 mM were growth inhibitory [16], and thus tests for photoautotrophic growth at concentrations above this were not performed.

The use of lactate as a carbon source by *Hph. fasciatum* is afforded by the presence of genes encoding both lactate permease and lactate dehydrogenase. In addition, genes encoding alcohol dehydrogenase, aldehyde dehydrogenase, and acetyl-CoA synthetase were all identified in the *Hph. fasciatum* genome and presumably account for the utilization of ethanol as a growth substrate, an ability shared with *Hmi. gestii*. Based on the presence of several key genes (e.g., those genes encoding coenzyme A transferase, acyl-(butyryl)-CoA dehydrogenase, enoyl-CoA hydratase, 3-hydroxybutyryl-CoA dehydrogenase, and acetyl-CoA C-acetyltransferase), butyrate metabolism in *Hph. fasciatum* appears to be identical to that described in *Hrs. convoluta* [13].

Although photoassimilation of propionate was not tested in the species description of *Hph. fasciatum*, the ability of this species to use propionate as a carbon source seems likely because genes encoding propionyl-CoA carboxylase, methylmalonyl-CoA epimerase, and methylmalonyl-CoA mutase were all identified in the *Hph. fasciatum* genome. This genetic profile matches that of *Hrs. convoluta* and *Hrs. acidaminivorans*, both of which photoassimilate and grow photoheterotrophically on propionate (notably, this synteny was also observed in the genomes of *Hmi. modesticaldum* and *Hmi. gestii*, species that have not been shown to utilize propionate but may also be able to do so). It is likely that this same gene complement also occurs in *Hrs. daurensis*, a species in which growth on propionate was also observed [25] but for which no genome sequence is available. Although propionate did not support photoheterotrophic growth of either *Hrs. baculata* [26] or *Hmi. sulfidophilum* [27], it did serve as a suitable carbon source for most species of *Heliorestis*, as well as *Hmi. undosum* [27]. Considering the prevalence of these genes in heliobacteria, propionate assimilation may be more widespread among heliobacteria than previously thought.

According to culture-based studies, all neutrophilic heliobacteria can grow by pyruvate fermentation in darkness [4,5]. By contrast, alkaliphilic heliobacteria, including all species of *Heliorestis* and *Candidatus* “Heliomonas lunata” (Figure 1), cannot grow in darkness and thus appear to be obligate photoheterotrophs. Depending on the species of heliobacterium, pyruvate is fermented with or without the production of H_2_ [28], suggesting that pyruvate is metabolized by either pyruvate:ferredoxin oxidoreductase (H_2_ produced) [29] or pyruvate:formate lyase (no H_2_ produced). Genes encoding pyruvate:ferredoxin oxidoreductase are present in the *Hph. fasciatum* genome, although the biochemistry of pyruvate fermentation in this species has not been studied. However, the absence of key genes encoding a H_2_-evolving [Fe-Fe] hydrogenase in all genome-sequenced *Heliomicrobium* species (Table 2) and in *Hph. fasciatum* suggests that these species do not evolve H_2_ while fermenting pyruvate. Curiously, however, as in species of *Heliomicrobium* [5], the genome of *Hph. fasciatum* contained a homolog of *hydA*, which encodes the H_2_-evolving HydA polypeptide of the [Fe-Fe] hydrogenase, but lacked the *hydEFG* maturase genes required to synthesize a functional enzyme. By contrast, both *hydA* (two copies) and *hydEFG* are present in the genomes of *Hbt. chlorum* and *Hbt. mobile* and the latter species evolves H_2_ during pyruvate fermentation [28]. Surprisingly though, *Hrs. convoluta* and *Hrs. acidaminivorans*—alkaliphiles that do not ferment pyruvate—also possess two copies of *hydA* and one copy of *hydEFG*. Thus, the distribution (or indeed, the very existence) of functional [Fe-Fe] hydrogenases in heliobacteria requires further study. In contrast to an [Fe-Fe] H_2_-evolving hydrogenase, genes encoding a [Ni-Fe] uptake-type hydrogenase are present in the *Hph. fasciatum* genome and in all heliobacteria with sequenced genomes (Table 2).

Although the genome of *Hph. fasciatum* encodes no obvious mechanism for carbohydrate uptake, such as the ribose ABC transporter identified in *Hmi. modesticaldum* and *Hrs. convoluta* [10,13], genes encoding complete glycolytic and nonoxidative pentose phosphate pathways were identified. As is true for all other sequenced heliobacteria, genes encoding glucose 6-phosphate dehydrogenase and 6-phosphogluconolactonase were absent in *Hph. fasciatum*, suggesting that the possession of incomplete Entner–Doudoroff and oxidative pentose phosphate pathways is universal among the *Heliobacteriaceae*.

Genes encoding enzymes of a complete citric acid cycle (CAC) were identified in *Hph. fasciatum*. The unusual citrate (*re*)-synthase identified in the genomes of both *Hmi. modesticaldum* [10] and *Hrs. convoluta* [13] was also encoded in the *Hph. fasciatum* genome. Described in *Hmi. modesticaldum* by Tang et al. [17], this unorthodox form of citrate synthase may have ancestral lineage within nonphototrophic clostridia, in which the enzyme is also found [5], and is presumably a common feature of heliobacterial central carbon metabolism.

Like other heliobacteria, *Hph. fasciatum* lacks a gene encoding citrate lyase and is, therefore, incapable of autotrophic growth using the reverse CAC present in green sulfur bacteria. Likewise, key genes of the Calvin–Benson cycle are absent in the *Hph. fasciatum* genome. Although a gene homologous to the C-terminal domain of the ribulose-bisphosphate carboxylase (RuBisCO) large subunit (*cbbL*) was identified in the genomes of *Hph. fasciatum*, *Hbt. mobile*, *Hbt. chlorum*, and *Hmi. undosum*, (but not in the genomes of *Hmi. modesticaldum*, *Hmi. gestii*, *Hrs. convoluta*, and *Hrs. acidaminivorans*), no gene encoding the corresponding small subunit of RuBisCO (*cbbS*) was identified. Moreover, as is the case with other heliobacteria, no gene encoding phosphoribulokinase was present in the genome of *Hph. fasciatum*.

Presumably, anaplerotic CO_2_ assimilation through the activity of phosphoenolpyruvate (PEP) carboxykinase (encoded by *pckA*) is an important mechanism of non-autotrophic CO_2_ fixation for all heliobacteria [5,17]. Interestingly, however, although *pckA* is present in the genomes of all other heliobacteria for which a sequence is available, no homolog encoding PEP carboxykinase could be identified in the genome of *Hph. fasciatum*. By contrast, as previously mentioned, the *Hph. fasciatum* genome does encode pyruvate:ferredoxin oxidoreductase, and in addition to evolving CO_2_ during pyruvate fermentation, this enzyme can assimilate CO_2_ by carboxylating acetyl-CoA to pyruvate. To replenish CAC intermediates drawn off for biosynthetic purposes in *Hph. fasciatum*, oxaloacetate can be synthesized from pyruvate using an encoded ATP-dependent pyruvate carboxylase.

Nearly all heliobacteria require biotin as a growth factor, with the only documented exceptions being *Hrs. convoluta* and *Hrs. acidaminivorans* [12,14]. The genomes of both of these species contain the full complement of biotin biosynthesis genes (*bioABCDF*). By contrast, the genomes of most other sequenced species of heliobacteria contain *bioABD* but lack genes encoding BioC (malonyl-[acyl-carrier protein] O-methyltransferase) and BioF (8-amino-7-oxononaoate synthase), enzymes required for biotin biosynthesis. Among sequenced heliobacteria, *Hph. fasciatum* is uniquely deficient in regard to biotin biosynthesis, as its genome contains *bioB*, which encodes biotin synthase but lacks all other genes of the biotin (*bio*) operon.

### 3.4. Nitrogen Metabolism: Utilization of Fixed Nitrogen

*Heliophilum fasciatum* grows using ammonia, glutamine, yeast extract, or dinitrogen as nitrogen sources; glutamate or aspartate are not used [16]. Two separate and divergent genes encoding ammonium transporter (Amt) proteins are present to import ammonia. Genes encoding the common glutamine synthetase–glutamate synthase pathway are also present, allowing for the incorporation of ammonia into key organic nitrogen compounds. In addition, a gene encoding the substrate-binding domain of an ABC glutamine transporter is present and may account for the organism’s ability to use externally supplied glutamine. While *Hph. fasciatum* has not been shown to use nitrite as a nitrogen source, and nitrite transporters have not been identified in any heliobacterium [5], a NrfAH-type nitrite reductase is encoded in the *Hph. fasciatum* genome. By contrast, genes encoding an assimilative nitrate reduction system could not be identified.

### 3.5. Nitrogen Metabolism: Nitrogen Fixation

**Figure 3:**
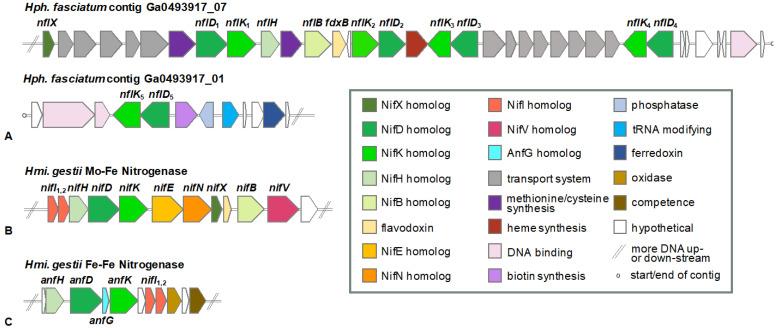
Genetic organization of nitrogen fixation-related genes and surrounding genes. (**A**) *Hph. fasciatum* loci containing genes encoding the nitrogen fixation-like homologs NflDK_1–5_. The following JGI/IMG coordinates are illustrated: Ga0493917_07: 106,630 to 145,155 and Ga0493917_01: 75 to 11,134; (**B**) *Hmi. gestii* locus containing genes encoding the Mo-Fe nitrogenase (NifHDK). The following Genbank coordinates are illustrated: NZ_WXEX01000020.1: 13,816 to 26,039; (**C**) *Hmi. gestii* locus containing genes encoding the alternative Fe-Fe nitrogenase (AnfHDGK). The following Genbank coordinates are illustrated: NZ_WXEX01000005.1: 245,244 to 257,234. Predicted functions are described in the key.

Although the genomics supporting the utilization of fixed nitrogen compounds by *Hph. fasciatum* is not unusual, such is not true of the genomics supporting its diazotrophic growth. A nitrogenase system has been detected by acetylene reduction in N_2_-grown cells of *Hph. fasciatum*, albeit activities are expressed at lower levels than in cells of *Heliomicrobium gestii* or *Heliomicrobium mobile* [15,16]. Six-genes encoding NifHDK (dinitrogenase reductase and the α- and β-subunits of dinitrogenase, respectively) and NifENB (nitrogenase assembly and maturase proteins) are present in virtually all diazotrophs that produce a FeMo-cofactor-containing nitrogenase and are considered the minimal gene set required for a diazotrophic phenotype [30]. Curiously, however, experiments to detect genes encoding dinitrogenase reductase (*nifH*) and the α subunit of dinitrogenase (*nifD*) in *Hph. fasciatum* by Southern blot and PCR analyses were unsuccessful, suggesting that a unique enzyme or pathway for nitrogen fixation might exist in this phototroph [31]. The *Hph. fasciatum* genome supports this hypothesis by revealing genes encoding five distinct NifDK-like proteins that contain the pfam 00148 oxidoreductase domain present in the α- and β-subunits of all known FeMo-cofactor-containing dinitrogenases [30]. All genes encoding these *n*itrogen *f*ixation-*l*ike proteins in *Hph. fasciatium* (genes and proteins abbreviated *nfl* and Nfl, respectively, and numbered 1–5) are clustered, with genes encoding NflDK_1–4_ located at the end of the contig Ga0493917_07 and those encoding NflDK_5_ positioned near the beginning of contig Ga0493917_01 (Figure 3A). In contrast to this arrangement, a “traditional *nif* cluster” containing *nifI*_1_, *nifI*_2_, *nifH*, *nifD*, *nifK*, *nifE*, *nifN*, *nifX*, *fdxB*, *nifB*, and *nifV* present in the genomes of *Hmi. modesticaldum* [10], *Hrs. convoluta* [13], and *Hmi. gestii* (Figure 3B), is absent from the *Hph. fasciatum* genome. Instead, single copies of *nfl* genes similar to *nifB*, *nifH*, *nifX*, and *fdxB* are present in the same chromosomal locus in *Hph. fasciatum* as genes encoding NflDK_1_ (Figure 3A). A NifV (homocitrate synthase) homolog is encoded elsewhere in the *Hph. fasciatum* chromosome; however, homologs of NifE and NifN could not be identified. Additionally, genes encoding an alternative nitrogenase, such as the FeFe-cofactor nitrogenase of *Hmi. gestii* (Figure 3C), were also absent from *Hph. fasciatum*.

The apparent absence of genes encoding NifEN in *Hph. fasciatum* is of particular interest but is not without precedence. FeMo nitrogenases lacking NifE or NifN (or both) are thought to be “ancestral nitrogenases”, enzymes that employ a truncated cofactor assembly pathway and catalyze an activity in addition to N_2_ reduction [30,32]. For example, the diazotrophic methanogen *Methanocaldococcus* sp. strain FS406-22 and bacterium *Caldicellulosiruptor* sp. strain YA01 synthesize nitrogenases that lack NifN but still contain NifE [33,34]. However, the genome of *Endomicrobium proavitum* strain Rsa215, a diazotrophic free-living termite gut bacterium, is missing genes encoding both NifE and NifN [35] indicating that a functional nitrogenase can be synthesized that lacks these proteins.

**Figure 4:**
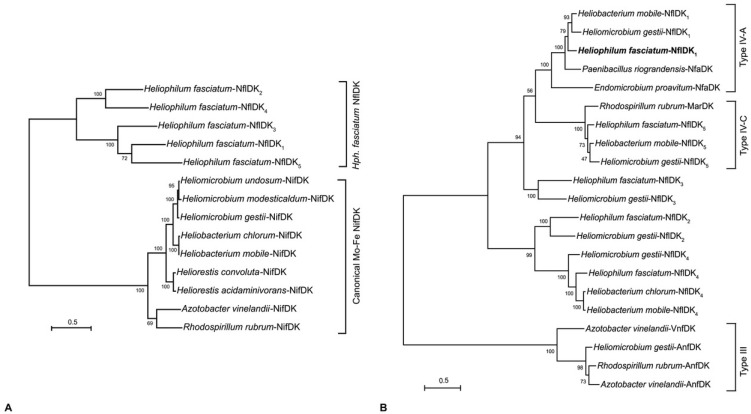
Maximum-likelihood phylogenetic trees of *Hph. fasciatum* concatenated NflDK sequences. (**A**) Comparison with canonical Mo-Fe nitrogenase NifDK sequences. (**B**) Comparison with Type III and IV nitrogen fixation-like sequences. The nodes represent bootstrap values based on 100 replicates, and scale bars indicate 0.5 changes per position. Taxa accession numbers are as follows with those beginning with a number corresponding to the JGI/IMG database and those beginning with a letter from Genbank: *Hph. fasciatum*—NflDK_1_ (2929298242, 2929298243), NflDK_2_ (2929298250, 2929298249), NflDK_3_ (2929298253, 2929298252), NflDK_4_ (2929298263, 2929298262), NflDK_5_ (2929297118, 2929297117); *Hmi. gestii*—NifDK (WP_161263156, WP_161263155), AnfDK (WP_161261510, WP_161261560), NflDK_1_ (WP_161260453, WP_161260452), NflDK_2_ (WP_161260457, WP_161260458), NflDK_3_ (WP_161260450, WP_161260449), NflDK_4_ (WP_161260444, WP_161260443), NflDK_5_ (WP_161260462, WP_161260461); *Hmi. modesticaldum*—NifDK (641558455, 641558456); *Hmi. undosum*—NifDK (WP_161255976, WP_161255978); *Hrs. acidaminivorans*—NifDK (2914075535, 2914075536); *Hrs. convoluta*—NifDK (QGG48552, QGG48553); *Hbt. chlorum*—NifDK (WP_188040647, WP_188040648), NflDK_4_ (WP_188039480, WP_188039481); *Hbt. mobile*—NifDK (WP_155476316, WP_155476317), NflDK_1_ (WP_155475363, WP_155475362), NflDK_4_ (WP_155475372, WP_155475371), NflDK_5_ (WP_155475353, WP_155475354); *A. vinelandii*—NifDK (643803063, 643803064), AnfDK (643807739, 643807737), VnfDK (643803186, 643803184); *Rsp. rubrum*—NifDK (637825100, 637825101), AnfDK (637825484, 637825482), MarDK (637824885, 637824884); *E. proavitum*—NfaDK (WP_052570612, WP_052570613); *P. riograndensis*—NfaDK (WP_020429361, WP_046504163).

Because the NifEN proteins are hypothesized to be encoded by genes originating from a *nifDK* duplication [36], the *Hph. fasciatum* NflDK_1–5_ homologs (Figure 3A) were compared to NifDK and NifEN homologs from other heliobacteria as well as from the model diazotrophs *Azotobacter vinelandii* and *Rhodospirillum rubrum*. The results showed that none of the five *Hph. fasciatum* NflDK proteins formed a clade with NifEN homologs (data not shown) or with NifDK (Figure 4A). Thus, despite possessing genes encoding molybdate transport (ModA) and molybdenum cofactor synthesis (Moa/Moe), *Hph. fasciatum* is the only known species of heliobacteria to lack both NifEN and an ortholog of the classical NifDK nitrogenase.

A search for NflDK_1–5_ homologs in other species of heliobacteria identified two such proteins in *Hbt. chlorum*, four in *Hbt. mobile*, and five in *Hmi. gestii* (Figure 4B). In addition to the five *Hmi. gestii* NflDK homologs, the *Hmi. gestii* genome encodes a classical NifDK as well as a homolog of the Fe-Fe alternative dinitrogenase (AnfDK) of *Azotobacter vinelandii* and *Rsp. rubrum* (Figure 3B,C and Figure 4A,B) (alternative nitrogenases lack Mo but contain Fe-Fe or V-Fe cofactors and are thought to be redundant enzymes that support nitrogen fixation when Mo is limiting [37]). Our discovery of *anfDK* in *Hmi. gestii* (Figure 3C) complements a previous finding of an *anfH*-like gene in this species [32] and physiological evidence for an Fe-Fe nitrogenase system [38]. Moreover, an examination of the chromosomal locus encoding AnfHDK in *Hmi. gestii* also indicated the presence of a gene encoding the δ-subunit (*anfG*) of its Fe-Fe nitrogenase (Figure 3C).

Phylogenetic studies of nitrogenases have revealed at least six groups (designated I–VI) of “*nif*-like” genes distributed throughout *Bacteria* and *Archaea*, with members of groups IV–VI predicted to encode alternative functions such as roles in (bacterio)chlorophyll biosynthesis or archaeal methyl coenzyme-M reductase cofactor F_430_ biosynthesis, as well as various aspects of sulfur metabolism [21,22,31,35,39,40,41,42,43]. Phylogenetic analysis of the *Hph. fasciatum* NflDK_1–5_ homologs indicates that they do not form a clade with Nif, Anf, or Vnf nitrogenases (groups I–III). Instead, the NflDK_1–5_ proteins are more similar to the group IV MarDK, NflDK, and NfaDK (*n*itrogen *f*ixation IV—subgroup *A*) proteins of *Rhodospirillum rubrum*, *Paenibacillus riograndensis*, and *Endomicrobium proavitum*, respectively [22,35] (Figure 4B). Whereas the MarDK proteins of *Rsp. rubrum* do not function as a nitrogenase [22], and the role of the NfaDK proteins in *P. riograndensis* is unknown (*P. riograndensis* also possesses canonical Nif and alternative Anf nitrogenases) [44], the *E. proavitum* NfaDK homolog falls within subgroup IV-A and is predicted to possess all the ligands required for N_2_ reduction [35]. Moreover, despite lacking homologs to NifEN, cells of *E. proavitum* incorporate ^15^N_2_ into biomass, presumably through the activity of NfaDK, the only *nif*-like genes present in the genome [35]. Therefore, based on its placement in a clade with *E. proavitum* NfaDK (Figure 4B) and the absence of genes encoding a classical nitrogenase system (Figure 4A), we predict that diazotrophy in *Hph. fasciatum* is supported by its NfaDK homolog NflDK_1_. If true, and if such an enzyme is a less robust nitrogenase than are classical nitrogenases, this could explain the lower nitrogenase activities observed in cells of *Hph. fasciatum* compared with those of other heliobacterial species [15,16].

**Figure 5:**
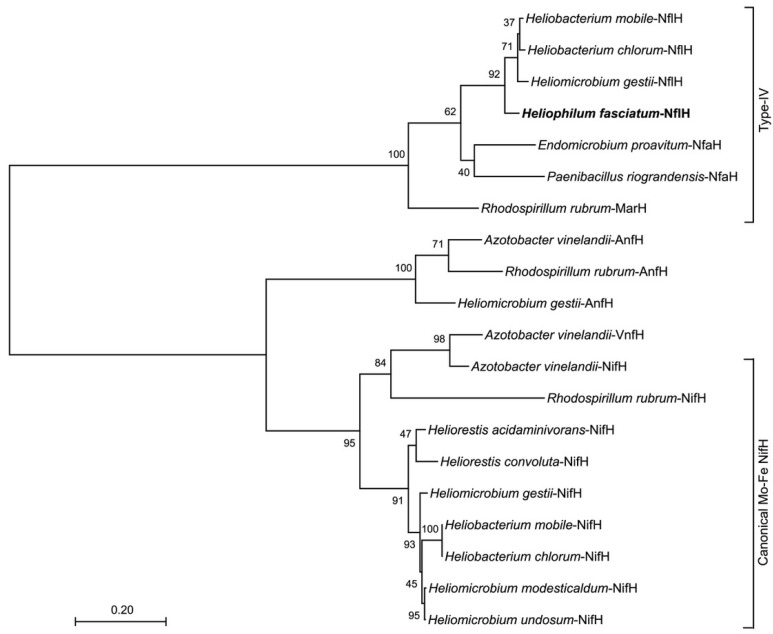
Maximum-likelihood phylogenetic tree of the *Heliophilum fasciatum* NflH sequence. The nodes represent bootstrap values based on 100 replicates and the scale bar indicates 0.2 changes per position. Taxa accession numbers are as follows with those beginning with a number corresponding to the JGI/IMG database (those beginning with a letter are from Genbank): *Hph. fasciatum*—NflH (2929298244); *Hmi. gestii*—NifH (WP_161263157.1), AnfH (WP_170294419), NflH (WP_161260455); *Hmi. modesticaldum*—NifH (641558454); *Hmi. undosum*—NifH (WP_161255974.1); *Hrs. acidaminivorans*—NifH (2914075534); *Hrs. convoluta*—NifH (QGG48551.1); *Hbt. chlorum*—NifH (WP_155476315.1), NflH (WP_188039485.1); *Hbt. mobile—*NifH (WP_155476315.1), NflH (WP_155475351.1); *A. vinelandii*—NifH (643803062), AnfH (643807740), VnfH (643803191); *Rsp. rubrum*—NifH (637825099), AnfH (637825485), MarH (637824886); *E. proavitum*—NfaH (WP_052570620.1); *P. riograndensis*—NfaH (WP_020429374.1).

While the roles of *Hph. fasciatum* NflDK_2–4_ are unknown, it is notable that the *Hph. fasciatum, Hmi. gestii*, and *Hbt. mobile* NflDK_5_ homologs form a clade with the *Rhodospirillum rubrum* MarDK proteins of the MarBHDK system that synthesizes methionine from volatile organic sulfur compounds (VOSCs) such as dimethyl sulfide and (2-methylthio)ethanol [22] (Figure 4B). MarDK are group IV-C nitrogenase-like proteins encoding the methylthio-alkane reductase that cleaves the C–S bond of VOSCs. Utilization of the MarBHDK pathway to assimilate sulfur from organic sources may compensate for the apparent lack of a sulfite reduction system in *Hph. fasciatum* (see Section 3.5). *Hmi. gestii*, *Hbt. mobile*, and *Hbt. chlorum* also possess an additional *nifH*-like (*nflH*) gene linked to the genes encoding these MarDK homologs. These NifH homologs form a clade with the MarH protein of *Rsp. rubrum* (Figure 5) and likely encode the electron-delivering subunit of a methylthio-alkane reductase. *Hph. fasciatum* possesses only one *nifH*-like gene, and it lies adjacent to the genes encoding NflDK_1_ in the genome (Figure 3A). Surprisingly, however, this NifH-like protein along with NfaH proteins of *P. riograndensis* and the *E. proavitum* nitrogenase form a clade with MarH instead of with NifH from other heliobacteria or AnfH or VnfH of Fe-Fe and V-Fe nitrogenases (Figure 5). All of these proteins possess the same critical residues necessary for reductive activity, which may allow for their cross-reactivity.

How the *Hph. fasciatum* and *E. proavitum* genomes have come to share these divergent NifHDK homologs is puzzling. Whereas both microbes are obligate anaerobes and fermentative, one is phototrophic while the other is symbiotic, and thus their ecology is unrelated. Why *Hph. fasciatum* is the only known heliobacterium to lack a conventional nitrogenase is also an intriguing question. Although we acknowledge that our analysis is not based on a single-contig closed genome, we nevertheless feel, for three reasons, that genes encoding a canonical nitrogenase system in *Hph. fasciatum* are truly absent. First, sequencing coverage of the *Hph.* *fasciatum* genome was high (477X), thus reducing the chance of losing or overlooking multi-gene operons. Second, experiments by others using PCR and Southern blots to detect *nifH* and *nifD* in *Hph.* *fasciatum* were negative, although both genes were easily identified in other heliobacterial species [31]. Finally, using degenerate primers targeting *nifD*, *anfD*, and *vnfD* [45], no PCR amplicons were obtained from *Hph. fasciatum* genomic DNA, although a canonical *nifD* was obtained from two other heliobacterial species used as controls (K.S.B., unpublished results).

Another interesting aspect of the *Hph. fasciatum* nitrogenase story is the lack of genes encoding obvious nitrogen metabolism regulators. A gene encoding a P-II nitrogen regulatory protein is present upstream of a gene encoding an ammonia transporter (2929297543), but this is not close to the loci encoding the NflDK_1–5_ proteins. Further studies are thus needed to determine the expression patterns of the *Hph. fasciatum* NflDK_1–5_ homologs, as well as mutational and ^15^N_2_ incorporation experiments to confirm the nitrogenase activity of *Hph. fasciatum* NflDK_1_ and test the N_2_-reducing ability of mutant derivatives lacking one or more homologs. The significance of encoding multiple NifDK-type proteins is also unknown, but it is not unusual for genomes encoding group IV nitrogenase-like enzymes to contain multiple copies of *nifDK*-like genes [43]. It is therefore possible that these genes encode important biochemical functions that are yet to be recognized.

### 3.6. Sulfur Metabolism

Unlike many heliobacteria that require a reduced source of sulfur for biosynthetic purposes [4,5,9], *Hph. fasciatum* grows in defined media with sulfate as the only sulfur source [16]. In this regard, the *Hph. fasciatum* genome encodes the widely distributed CysPTWA-mediated sulfate uptake system: CysNDC proteins that make adenylphosphosulfate (APS) and phosphoadenylphosphosulfate (PAPS) from sulfate, and AprAB and CysH that make sulfite from APS and PAPS, respectively. However, the *Hph. fasciatum* genome lacks homologs of CysJI, the classical alpha-beta sulfite reductase that reduces sulfite to sulfide in a wide variety of bacteria. Two genes were present that encoded a protein weakly similar to CysI (beta subunit of sulfite reductase). These genes (IDs 292929318 and 2929297321), were nearly identical and were annotated as “sulfite reductase beta subunit-like” but were only 20% identical to authentic CysI from *Escherichia coli* or *Bacillus subtilis*. Although it is possible that the *Hph. fasciatum* CysI-like protein participates in sulfite reduction, the absence of a homolog encoding the alpha subunit (CysJ) of sulfite reductase leaves open the question of exactly how sulfite reduction occurs in *Hph. fasciatum*.

Most heliobacteria will grow in media containing sulfide, and some species are remarkably sulfide tolerant [9,14]. *Hph. fasciatum* is an exception and is completely growth inhibited by as little as 0.1 mM sulfide [16]. Consistent with this, *Hph. fasciatum* lacks the common sulfide oxidation system—sulfide:quinone oxidoreductase—of purple and green sulfur bacteria [46]. In addition, *Hph. fasciatum* lacks *sox* genes (as do all known heliobacteria), which encode enzymes necessary to oxidize thiosulfate, the other reduced inorganic sulfur compound commonly oxidized as an electron donor by anoxygenic phototrophs. Thus, once sulfide is available in the *Hph. fasciatum* cell, from either the reduction of sulfate or from organic sources, it is likely to be quickly incorporated into sulfur-containing amino acids. In this regard, *Hph. fasciatum* is well equipped. Its genome encodes cysteine synthase that makes cysteine from serine and sulfide, as well as all genes necessary to convert aspartate to homocysteine, from which methionine is made. In addition, the genome encodes an ABC transporter for methionine and an ABC system for the uptake of branched-chain amino acids. However, as for methionine, all *Hph. fasciatum* genes necessary for the biosynthesis of valine, leucine, and isoleucine could be identified.

### 3.7. Motility

Cells of *Hph. fasciatum* are large rods (Figure 2) that display an unusual form of motility, unique among heliobacteria. *Hph. fasciatum* cells form bundles of parallel cells resembling the rafts of *Bacillus* or *Proteus* that form in biofilms [47,48]; the *Hph. fasciatum* bundles are motile as a unit and move with a rolling motion [16]. Bacterial flagella consist of three major components: the basal body, which traverses the cytoplasmic membrane (and if present, the outer membrane), the hook complex, and the filament, the rotating component that imparts cell motility [49]. Although both gram-positive and gram-negative bacterial flagella contain a basal body and hook that connects with the filament, gram-positive species lack the L and R rings that imbed the flagellum into the outer membrane of gram-negative bacteria [50]. In agreement with the *Bacillus subtilis* genome, only two of the three Flh proteins (FlhAB), highly conserved proteins required for exporting flagellar components across the cytoplasmic membrane [51], were identified in the *Hph. fasciatum* genome. A gene encoding FlhE, a protein found primarily in gram-negative enteric bacteria that functions to prevent proton leakage during the secretion of flagellar components across the cytoplasmic membrane, is missing, but it has been shown to be unessential for swimming motility [52].

Genes encoding Flg proteins (responsible for forming or regulating the formation of the flagellar rod, P ring, and hook) [53,54] in the *Hph. fasciatum* genome also followed the *Bacillus* pattern; genes encoding all eight of the Flg proteins of *Bacillus* (FlgBCDEFGKLMN) were identified. In addition, an unidentified protein with significant homology to FlgJ, a protein missing in *Bacillus*, was identified in the *Hph. fasciatum* genome and shared 50–60% identity with FlgJ from several gram-positive bacteria. FlgJ is a peptidoglycan hydrolase [54] and is found in the genomes of all heliobacteria listed in Table 2. Flg proteins not encoded in the *Hph. fasciatum* genome include FlgAHI and FlgOPQT, outer membrane proteins or proteins otherwise unessential for motility in gram-positive bacteria [53,54].

Genes encoding most Fli proteins responsible for forming the basal body, hook, filament, and cytoplasmic flagellar components (FliACDEFGHIJKLMNOPQRS) [53,54] were identified in the *Hph. fasciatum* genome, but the filament chaperone protein FliT, unessential in many motile bacteria, and regulatory proteins FliZY showed less than 50% identity to homologs from *Bacillus*. Mot proteins form the stator complex in the flagellar basal body [53], and the *Hph. fasciatum* genome encodes MotA and MotB, as does *Bacillus*. As expected, MotCD, proteins that form a second stator in *Pseudomonas aeruginosa* and some other gram-negative bacteria, and MotXY, proteins that form an alternative stator in bacteria that use a Na^+^-motive force instead of a H^+^-motive force to power the flagellum, such as *Vibrio alginolyticus*, were not encoded in the *Heliophilum* genome.

The *Heliophilum* genome encoded several chemotaxis proteins, suggesting that the bundled cells can respond to chemical attractants and repellants. Chemotaxis-specific proteins included virtually all “Che” proteins (CheABCDRWXY) plus at least five chemical sensors, the methyl-accepting chemotaxis proteins [55]. The only major *Escherichia coli* Che gene not identified in *Hph. fasciatum* was CheZ. This protein has phosphatase activity and functions to dephosphorylate CheY, the protein that controls the direction of flagellar rotation. CheZ is distributed only in certain *Proteobacteria*, and its function is replaced in gram-positive bacteria by CheC and CheX [56], both of which were encoded in the *Hph. fasciatum* genome.

Although no obvious answer emerged to explain the bundle-forming phenotype of *Hph. fasciatum*, this behavior could be a manifestation of biofilm formation. In this connection, cultures of *Hph. fasciatum* streaked on agar plates form spreading, diffuse colonies [16]. Rafts of cells are common in biofilm-forming bacterial species, and the dynamic nature that exists between free-swimming individual cells and rafts of cells indicates that specific “glue-like” substances are not required to form rafts [47]. Instead, it is likely that rafting is the product of intercellular bundling of flagella to yield cells with their flagella interwoven in phase [47,57]; notably, such flagellar aggregates were observed in the original description of *Hph. fasciatum* [16]. From an ecological perspective, rafts might help *Hph. fasciatum* become established on a moist surface, such as a rice plant root, where individual cells could be washed away.

### 3.8. Sporulation

True to its phylogenetic roots with the endospore-forming *Firmicutes*, *Hph. fasciatum* forms endospores—dormant cells highly resistant to physiochemical extremes [58]—complete with the signature molecule of these structures, dipicolinic acid–Ca^2+^ complexes [16]. Moreover, experiments to hybridize *Hph. fasciatum* DNA with endospore-specific gene probes from *Bacillus subtilis* confirmed the genetic capacity for this phototroph (and several other species of heliobacteria) to produce endospores [6].

Over 500 gene products have been linked to endospores in *Bacillus subtilis*, several of which are unessential for endospore production [59]. Sporulation-specific genes in *Hmi. modesticaldum* were found to be but a small subset of those found in *B. subtilis*, although several key sporulation genes were identified. As in *Hmi*. *modesticaldum* and *Hrs*. *convoluta*, all five sigma factors absolutely essential for the formation of endospores (sigma E, F, G, H, and K) were identified in the *Hph. fasciatum* genome. An ortholog of the master transcription factor Spo0A, active during the early stages of sporulation [60], is present in all heliobacterial genomes, including that of *Heliophilum*. However, Spo0M, a key protein that regulates several genes essential for sporulation, is absent from the *Hmi. modesticaldum* and *Hrs. convoluta* genomes [10,13]. By contrast, the *Hph. fasciatum* genome contained a putative *spo0M* gene (30–40% identity to *spo0M* from several sporulating bacteria). *Hbt. chlorum*, *Hmi. gestii*, and *Hmi. undosum* but not *Hbt. mobile* also contained a *spo0M* homolog, although all of these species encoded proteins less than 40% identical to the *Hph. fasciatum* protein, suggesting considerable divergence in this protein in those heliobacteria that encode it.

Also present in the *Hph. fasciatum* genome were genes encoding a variety of small acid-soluble spore proteins (SASPs). These proteins function to protect DNA in endospores from damage by desiccation, ultraviolet radiation, and to some degree, from heat [60,61,62]. Genes encoding the major alpha/beta-type SASPs (*sspA* and *sspB*) were present and showed high homology to genes from all other heliobacteria and chemotrophic endospore-forming bacteria. Three different α/β-type proteins were found in the *Hph. fasciatum* genome compared with just two in the genomes of the two alkaliphiles, *Hrs. convoluta* and *Hrs. acidaminovorans*, and four in each of the other five genome-sequenced heliobacterial species that grow optimally at neutral pH (Figure 1). In addition, the *Hph. fasciatum* genome encoded a minor SASP found in *B. subtilis* but not encoded in any other heliobacterial genome. This SASP was of the “thioredoxin type” (*B. subtilis sspT*), a protein encoded only in the genomes of a subset of sporulating bacteria [61]. Although the function of this minor SASP is unknown, it is clearly an endospore-associated protein because its transcription is dependent on the spore-specific sigma factor, σF. The genomes of all heliobacteria other than *Hph. fasciatum* encoded a different minor SASP, *sspH*, a gene under the control of σK in the developing forespore and whose product is also of unknown function.

The endospore is covered by a coat consisting of both structural and enzymatic proteins, and in *B. subtilis*, these proteins are encoded by a variety of genes, including over 20 *cot* genes [63]. Although Cot proteins are widespread in *Bacillus* species, in *Clostridium* species many *cot* genes are missing [63]. The *Hph. fasciatum* genome contained a handful of *cot* homologs. CotJC was encoded in the genomes of all heliobacteria listed in Table 2 and is one of the few Cot proteins universally present in species of *Bacillus* and *Clostridium*. The inner spore core protein GerQ is also present in all heliobacterial genomes. Genes encoding the spore-coat proteins CotJA and CotJB were annotated in *Hph. fasciatum* but the proteins bore no close identity to *B. subtilis* CotJA and CotJB. Notably, however, the *Hph. fasciatum* CotJA and CotJB homologs bore significant similarities to CotJA/JB from *Clostridium perfringens*, suggesting that there may be different forms of these endospore-specific proteins in aerobes compared with anaerobes.

Finally, in contrast to all other heliobacteria, a homolog of CotSA, a protein universally distributed in species of *Bacillus* and *Clostridium* [63], was autoannotated in the *Hph. fasciatum* genome as a cell wall glycosyltransferase with ~30% identity to *B. subtilis* CotSA. A protein similar to the *B. subtilis* spore-coat protein CotF was also identified in the *Hph. fasciatum* genome but is absent from the genomes of other sequenced heliobacteria; CotF is a spore-coat protein widely distributed among endospore-forming bacteria [63]. Genes encoding homologs of the *B. subtilis* spore-coat proteins CotABCDEGHIKMNOPQRSUVWXYZ or CotIC, NE, NH, NW, and OO could not be identified in the *Hph. fasciatum* genome. Thus, as regards the genetics of sporulation, *Hph. fasciatum* has the essential genes necessary to form endospores but, like other heliobacteria with analyzed genomes, is missing many of the unessential genes present in the genome of the well-studied *Bacillus subtilis*.

## 4. Conclusions

The genome of *Heliophilum fasciatum* represents the first detailed analysis of a genome from a species of heliobacteria that grows optimally at moderate temperatures and neutral pH. Although no firm explanation emerged for the mechanism that allows this organism to move about its habitat in loosely attached bundles, the genomics supporting the physiology of this species revealed many things in common with other heliobacteria (e.g., lack of autotrophy, ability to grow in darkness by fermentation, production of endospores, to mention a few), as well as an important feature possibly unique to this species: diazotrophy in the absence of a canonical nitrogenase. Whether nitrogen fixation in the Tanzanian rice soil habitat of *Hph. fasciatum* [16] is best served by its unusual nitrogenase system remains to be seen. However, the fact that all other heliobacteria with sequenced genomes (some species of which originate from rice soils) contain a canonical nitrogenase (Figure 3, Figure 4 and Figure 5), suggests that the ancestor of *Hph. fasciatum* may have been incapable of diazotrophy but that eventually, this species acquired an enzyme with nitrogenase-like activity from either duplications or lateral transfers of genes encoding functionally related proteins. Nitrogen fixation by phototrophic purple bacteria in paddy soil environments is well known [64,65]. *Hph. fasciatum* and other paddy-dwelling heliobacterial species could also be contributing fixed nitrogen to rice plants, perhaps in exchange for organic compounds to fuel their photoheterotrophic metabolism.

## Figures and Tables

**Table 1:** Properties of *Heliophilum fasciatum* strain Tanzania^T^ compared with those of *Heliorestis convoluta* strain HH^T^ and *Heliomicrobium modesticaldum* strain Ice1^T^.

Property ^a^	Tanzania^T^	HH^T^	Ice1^T^
Cell morphology	Rods in bundles	Ring-shaped coils	Rods
Flagellar motility	Yes	Yes	Yes
Absorption maxima ^b^	792 nm	786 nm	788 nm
Characteristic carotenoid	4,4′-diaponeu-rosporene	OH-diaponeurosporene glucoside ester	4,4′diaponeu-rosporene
Growth temperature optima (°C)/pH optima	37/7	33/8.5−9	50−52/6.5
Photoassimilation of acetate or pyruvate	Yes	Yes	Yes
Other C sources photoassimilated	Lactate, Butyrate ^c^, ethanol ^c^	Propionate, butyrate	Lactate, glucose, fructose, ribose ^d^
Chemotrophic (dark, fermentative) growth	Yes	No	Yes
16S rRNA gene sequence identity to Tanzania^T^ (%)	100	90	93

^a^ data from [9,12,16]; ^b^ absorption maxima for bacteriochlorophyll *g* in intact cells suspended in anoxic 30% bovine serum albumin; ^c^ in the presence of bicarbonate; ^d^ weak growth on sugars compared to growth on pyruvate or lactate [17].

**Table 2:** Heliobacteria with sequenced genomes.

Genus/Species	Habitat/pH Optimum	Protein-Encoding Genes ^a^
*Heliobacterium chlorum*	Garden soil/7	3876 (NZ_JACVHF000000000.1)
*Heliobacterium mobile*	Paddy soil, Thailand/7	3699 (NZ_WNKU00000000.1)
*Heliomicrobium modesticaldum*	Hot springs, Reykjanes, Iceland/6-7 ^b^	2662 (NC_010337.2)
*Heliomicrobium gestii*	Paddy soil, Dar es Salaam, Tanzania/7	3266 (NZ_WXEX00000000.1)
*Heliomicrobium undosum*	Microbial mat, Garga Hot Springs, Siberia/7.5	3356 (NZ_WXEY00000000.1)
*Heliorestis acidaminivorans*	Lake Hamra, Wadi Natroun Egypt/9	2765 (NZ_WBXO00000000.1)
*Heliorestis convoluta*	Lake Hamra, Wadi, Natroun Egypt/8.5	2909 (NZ_CP045875.1)
*Heliophilum fasciatum*	Paddy soil, Dar es Salaam, Tanzania/7	^c^ 2834 (NZ_SLXT00000000.1)/2951

^a^ known from complete genomes of *Hmi. modesticaldum* [10] and *Hrs. convoluta* [13] and predicted from draft genome sequences of the other species. Genbank reference sequence numbers listed in parentheses; ^b^ strain dependent [9]; ^c^ number preceding the slash is from Genbank annotation; number following the slash is from this study.

**Table 3:** Comparative genome statistics for *Heliophilum fasciatum* Tanzania^T^, *Heliorestis convoluta*. HH^T^, and *Heliomicrobium modesticaldum* Ice1^T a^.

Characteristic	Tanzania^T^	HH^T^	Ice1^T^
Accession number	Ga043916 ^b^	CP045875 ^b^	CP000930 ^b^
Genome size (bp) ^c^	3,141,306	3,218,981	3,075,407
Contigs	75	1	1
Genome G+C (%)	50.9	43.1	56
Coding DNA (%)	95.7	90.1	90.6
Total ORFs	2951	3263	3138
Hypothetical proteins (%)	22.9	27.5	23.8
rRNAs	15	9	24
tRNAs	89	105	104
Average nucleotide identity (ANI)			
to genome of Tanzania^T^ (%) ^d^	100	66.7	68.8

^a^ data for strain HH^T^ taken from [13] and for strain Ice1^T^ from [10]; ^b^ for strain Tanzania^T^, this is the Gold Analysis Project ID, Joint Genome Institute Integrated. Microbial Genomes and Microbiomes (listed in the IMG as *Heliophilum fasciatum* MTM). For strain HH^T^ and strain Ice1^T^, these are accession numbers from Genbank; ^c^ each genome consists of a single chromosome with no plasmids. The genomes of strains HH^T^ and Ice1^T^ were each closed into a single contig and thus the genome size is precise. The strain Tanzania^T^ genome is the draft sequence from a total of 75 contigs; ^d^ data from [8].

## Data Availability

The *Heliophilum fasciatum* Tanzania^T^ genome sequence (listed as *Heliophilum fasciatum* MTM) is publically available in the JGI/IMG database (https://img.jgi.doe.gov/) under the genome ID 2929297113.
